# PI3K-PTEN dysregulation leads to mTOR-driven upregulation of the core clock gene BMAL1 in normal and malignant epithelial cells

**DOI:** 10.18632/oncotarget.9877

**Published:** 2016-06-07

**Authors:** Camila S. Matsumoto, Luciana O. Almeida, Douglas M. Guimarães, Manoela D. Martins, Petros Papagerakis, Silvana Papagerakis, Andreia M. Leopoldino, Rogerio M. Castilho, Cristiane H. Squarize

**Affiliations:** ^1^ Laboratory of Epithelial Biology, Department of Periodontics and Oral Medicine, University of Michigan School of Dentistry, Ann Arbor, MI, USA; ^2^ Comprehensive Cancer Center, University of Michigan, Ann Arbor, MI, USA; ^3^ Department of Orthodontics and Pediatric Dentistry, School of Dentistry, University of Michigan, Ann Arbor, MI, USA; ^4^ Center for Organogenesis, University of Michigan, Ann Arbor, MI, USA; ^5^ Department of Clinical Analysis, Toxicology and Bromatology, School of Pharmacy, University of Sao Paulo, Ribeirao Preto, SP, Brazil; ^6^ Department of Oral Pathology, School of Dentistry, University of Sao Paulo, SP, Brazil; ^7^ Department of Oral Pathology, School of Dentistry, Federal University of Rio Grande do Sul, Porto Alegre, RS, Brazil; ^8^ Department of Otolaryngology, Medical School, University of Michigan, Ann Arbor, MI, USA

**Keywords:** head and neck cancer, oral cancer

## Abstract

Dysfunctional clock signaling is observed in a variety of pathological conditions. Many members of the clock gene family are upregulated in tumor cells. Here, we explored the consequences of a commonly disrupted signaling pathway in head and neck cancer on the regulation of circadian clock genes. PTEN is a key molecular controller of the PI3K signaling, and loss of PTEN function is often observed in a variety of cancers. Our main goal was to determine whether PTEN regulates circadian clock signaling. We found that oxidation-driven loss of PTEN function resulted in the activation of mTOR signaling and activation of the core clock protein BMAL1 (also known as ARNTL). The PTEN-induced BMAL1 upregulation was further confirmed using small interference RNA targeting PTEN, and in vivo conditional depletion of PTEN from the epidermis. We observed that PTEN-driven accumulation of BMAL1 was mTOR-mediated and that administration of Rapamycin, a specific mTOR inhibitor, resulted in *in vivo* rescue of normal levels of BMAL1. Accumulation of BMAL1 by deletion of PER2, a Period family gene, was also rescued upon in vivo administration of mTOR inhibitor. Notably, BMAL1 regulation requires mTOR regulatory protein Raptor and Rictor. These findings indicate that mTORC1 and mTORC2 complex plays a critical role in controlling BMAL1, establishing a connection between PI3K signaling and the regulation of circadian rhythm, ultimately resulting in deregulated BMAL1 in tumor cells with disrupted PI3K signaling.

## INTRODUCTION

Head and Neck Squamous Cell Carcinoma (HNSCC) is one of the most common solid tumors worldwide. The risk factors for HNSCC are well known and include consumption of alcohol and tobacco and infection by HPV. Recent technological advances in large-scale sequencing resulted in the identification of common genetic alterations in HNSCC, including mutations occurring in genes of the phosphoinositide 3-kinase (PI3K) pathway [1–5], as *PIK3CA, GRB1, PIP5K3, AKT2, TSC1, TSC2, mTOR, Rictor, Raptor* among others. Loss of function and mutations of key molecular regulators of PI3K signaling, including PTEN, are associated with the formation of various solid tumors, such as head and neck cancer [1, 6–8]. *PTEN* is a tumor suppressor gene that negatively regulates PI3K signaling and downstream PI3K family members, including AKT and mammalian target of Rapamycin (mTOR). Interestingly, *PTEN* mutations and protein loss are common events in the carcinogenesis and progression of HNSCC and other cancers such as glioblastomas and cancers of the breast, endometrium, and prostate [1, 9–12]. In HNSCC, PTEN protein loss is close to 31% and may reach 50% in advanced cases [2, 6, 7, 13, 14] (Wagner and Squarize, under submission).

Although clinically relevant [7, 13], the mechanisms associated with loss of PTEN function are largely unknown. Epigenetic events are likely the primary cause of PTEN loss of function in solid tumors, such as malignant melanomas, sporadic breast cancer, hepatocellular carcinoma, and thyroid cancer [15–18]. Inactivation of PTEN protein also occurs following oxidative inactivation that is induced by H_2_O_2_. The H_2_O_2_ targets the redox active Cys^124^ residue of PTEN, resulting in the formation of a disulfide bond between Cys^71^ and Cys^124^, as described in cervix tumor cell lines and fibroblasts [19]. However, little is known about how oxidation affects PTEN signaling in HNSCC.

The aim of the study was to investigate the effects of compromised PTEN function mediated by the reduction/oxidation mechanism in the regulation of core clock genes. We found that oxidation of PTEN in HNSCC resulted in the accumulation of the core circadian molecule BMAL1 (i.e., ARNTL - Aryl hydrocarbon receptor nuclear translocator-like protein 1, Homo sapiens [Human], UniProtKB - O00327), which aligns to our studies in the epidermis of PTEN conditional knockout mice (Zagni *et al.,* submitted). Surprisingly, accumulation of BMAL1 was associated with upregulation of the mTOR pathway. *In vivo* inhibition of mTOR by Rapamycin causes reduced expression of BMAL1 in PTEN and Per2 knockout mice, two mouse models that constitutively express high levels of BMAL1 in the epidermis. Interfering with the mTOR pathway through siRNA technology aiming at Raptor and Rictor further strengthen our findings that mTOR plays a novel role in the control of the core clock gene BMAL1. These results suggest that pathological conditions leading to disruption of the PI3K/PTEN/mTOR axis are likely to deregulate the circadian clock in normal and epithelial-derived tumor cells.

## RESULTS

### PTEN oxidation in HNSCC activates mTOR signaling

**Figure 1:**
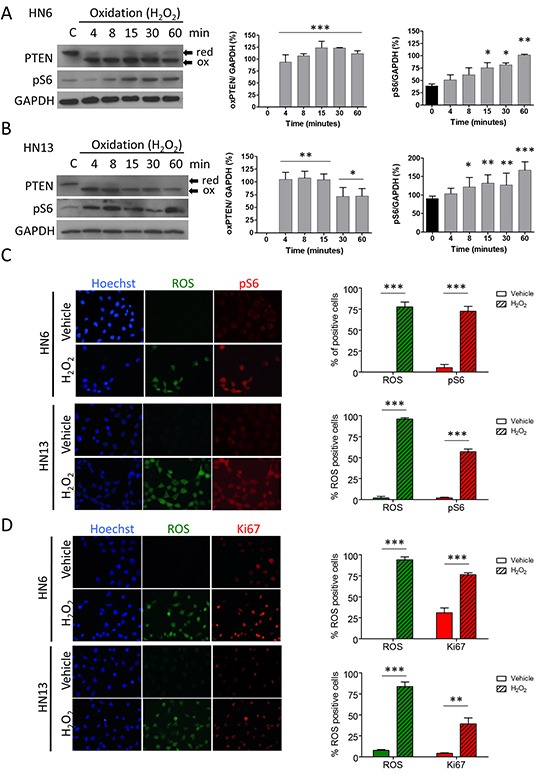
Oxidation of PTEN results in upregulation of mTOR signaling Oxidation of PTEN and pS6 activation is observed after of oxidative stress (H_2_O_2_) in HN6 **(A)** and HN13 **(B)** (*p<0.05, **p<0.01, ***p<0.001). **C.** Oxidative stress induces abrupt nuclear accumulation of reactive oxygen species (ROS) in head and neck cancer cell lines (green channel). Note that accumulation of ROS is accompanied by pS6 activation (red channel) (***p<0.001). **D.** Tumor cells oxidation leads to increased proliferation (Ki-67, red channel) and simultaneous accumulation of ROS (green channel)(**p<0.01, ***p<0.001).

Administration of hydrogen peroxidase caused oxidation of PTEN in HNSCC (Figure 1A and 1B; oxPTEN *p<0.05, **p<0.01, ***p<0.001), as previously described in HeLa and NIH 3T3 cells [19]. Interestingly, oxidation-mediated deactivation of PTEN resulted in time-dependent pS6 activation (Figure 1A and 1B; pS6 *p<0.05, **p<0.01, ***p<0.001), a molecular marker for mTOR activity and pharmacological inhibition efficacy [20]. Activation of pS6 becomes statistically significant after PTEN oxidation (Figure 1A and 1B). Used as a positive control or readout of oxidation, reactive oxygen species (ROS) was observed to build up on tumor cells along with the pS6 activation (Figure 1C, ***p<0.001) and the increased proliferation (Figure 1D, **p<0.01, ***p<0.001).

### Increased oxidation leads to the accumulation of the core clock protein BMAL1

**Figure 2:**
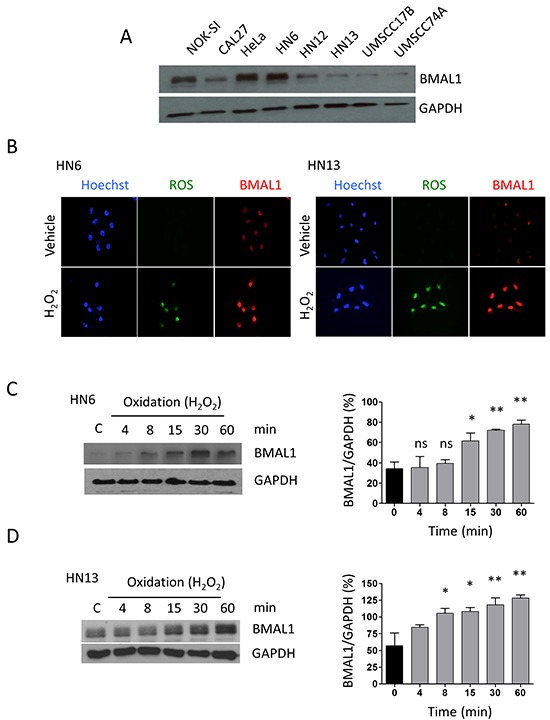
Oxidation causes accumulation of BMAL1 **A.** Head and neck cancer cells have different expression levels of the core clock protein BMAL1. **B.** Immunofluorescence assay depicts accumulation of ROS (green channel) and BMAL1 (red channel) upon oxidation. **C.** and **D.** Western blot assay demonstrates time-dependent accumulation of BMAL1 upon oxidative stress in HNSCC cells (*p<0.05, **p<0.01).

In the typical process, the cells in the body follow a circadian rhythm. The circadian rhythm oscillations orchestrate several biological processes intimately involved in the maintenance of tissue homeostasis and stem cell heterogeneity [21], and it may be dysregulated in cancer and aging. For example, disruption in the core clock protein BMAL1 results in severe premature aging [22] and overexpression is associated with decreased overall survival, particularly in colorectal cancer patients [23]. In cell culture, the circadian rhythm synchronizing agent Forskolin is a potent inducer of circadian gene expression including BMAL1 accumulation [24, 25]. Interesting, head and neck tumor cell lines present different levels of BMAL1 upon administration of forskolin. BMAL1 expression ranged from highly expressed protein observed in HN6 cells to extremely low levels as observed, for example, in HN13 cells (Figure 2A). We next examined whether exposure of HNSCC cells to H_2_O_2_ interferes with levels of BMAL1 in HNSCC cells. We found that administration of H_2_O_2_ resulted in the nuclear accumulation of BMAL1 in both HNSCC cell lines compared to vehicle, along with the accumulation of ROS (Figure 2B). Interestingly, HN13 cells, which had low levels of BMAL1 (Figure 2A), showed high accumulation of nuclear BMAL1 in response to oxidation (Figure 2B right panel). Accumulation of BMAL1 was also time dependent (Figure 2C and 2D)(*p<0.05, **p<0.01). Accumulation of BMAL1 in HN6 cells was statistically evident after 15 minutes of administration of H_2_O_2_ (Figure 2C) while HN13 cells showed statistical significance after 8 minutes of H_2_O_2_ administration (*p<0.05) (Figure 2D).

### Targeted disruption of PTEN in HNSCC results in activation of mTOR and accumulation of BMAL1

**Figure 3:**
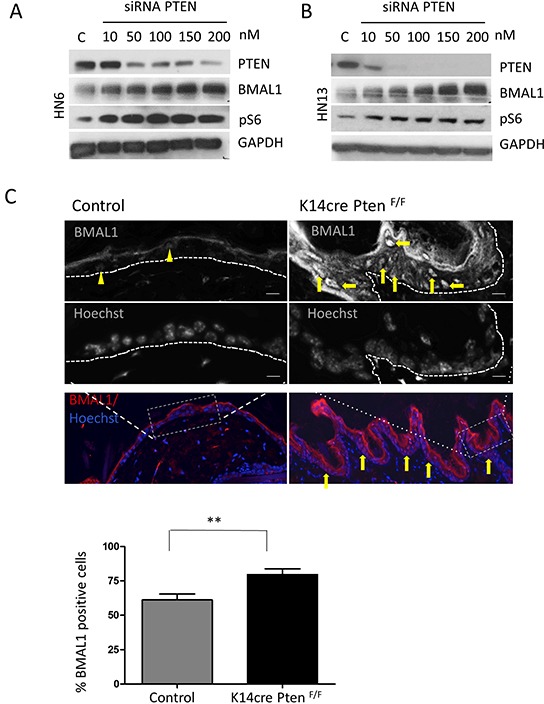
Targeted disruption of PTEN in vitro and in vivo induces activation of pS6 and BMAL1 **A.** and **B.** Targeted inhibition of PTEN using siRNA results in concentration-dependent inhibition of PTEN protein in HNSCC cells. HNSCC cells show accumulation of BMAL1 and pS6 in response to PTEN inhibition. **C.** Immunofluorescence assay to detect BMAL1 in PTEN conditional knockout mice (K14cre Pten^F/F^) and control littermates. Immunofluorescences and graphic show high accumulation of nuclear BMAL1 in K14cre Pten^F/F^ mice compared to control mice (K14cre). Scale bars represent 50 μm. Black and white images depict cells positive for BMAL1 in K14cre Pten^F/F^ mice (arrows) compared to few BMAL-positive cells in control mice (arrowhead). Scale bars represent 10 μm. (**p<0.01).

We next examined whether loss of PTEN function caused accumulation of BMAL1. PTEN-targeted siRNA (Figure 3A) (Figure 3B) resulted in a reduction of PTEN protein. PTEN suppression was accompanied by accumulation of BMAL1 in a siRNA concentration-dependent manner. Similarly, downregulation of PTEN resulted in the activation of mTOR signaling, as detected by the increase in pS6 (Figure 3A and 3B). The correlation between PTEN loss and activation of mTOR signaling is well documented by our group and others [20, 26–28]; however, concurrent inactivation of PTEN protein and upregulation of clock genes, such as BMAL1, is a novel discovery. It is unclear whether compromised PTEN function that leads to accumulation of BMAL1 is specific to head and neck cancer or is a normal regulatory mechanism found in epithelial cells. To address this question, we excised *PTEN* from epithelial cells *in vivo* using epithelial-specific *Pten* conditional knockout mice generated by crossing mice that harbor a floxed Pten allele containing two loxP sites (Pten^tm1Hwu^ or Pten^F/F^) with mice that express the Cre recombinase under the control of the K14 promoter (K14Cre)[20]. Through immunolocalization, we found that control mice present a mean of 61% of epithelial cells positive for nuclear BMAL1 localized in the interfollicular component of the epidermis (Figure 3C_Control arrowhead). Interestingly, deletion of PTEN from the basal layer of the epidermis resulted in *in vivo* accumulation of nuclear BMAL1 (mean 79%) in epithelial cells (Figure 3C_K14Cre Pten^F/F^, arrow). Of note, cornified layer of the epidermis (absent of nuclear structures) present typical unspecific staining for the secondary antibody. Collectively, our data suggest that *PTEN-*deficient HNSCC cells showing deregulated clock gene machinery can also be observed in normal epithelial cells presenting genetic excision of PTEN. These results suggest that PTEN/PI3K signaling pathway may play an unexpected role in the regulation of the core clock gene BMAL1 in epithelial cells.

### PTEN-mediated accumulation of BMAL1 requires activation of mTOR signaling

**Figure 4:**
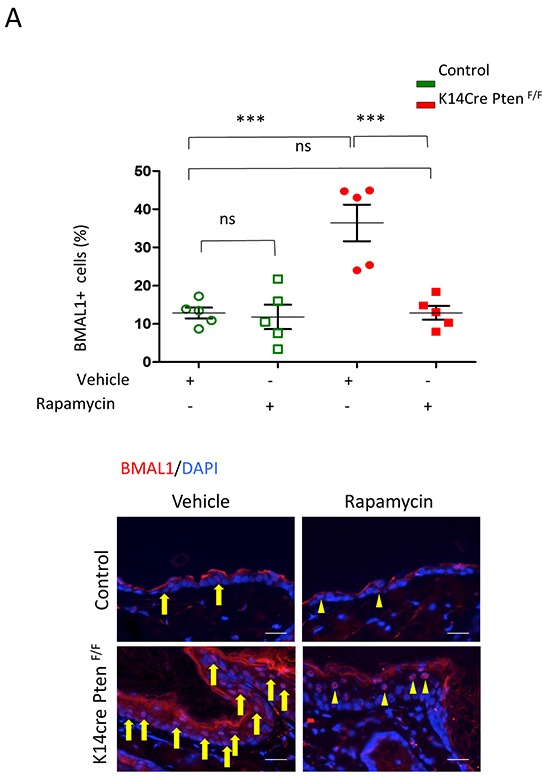
Inhibition of mTOR signaling with Rapamycin rescues PTEN-driven accumulation of BMAL1 **A.** Tissue samples and graphic show BMAL1 in K14cre Pten^F/F^ mice and control (arrows). Note a significant amount of BMAL1 expression in PTEN mutant mice. K14cre Pten^F/F^ mice and control littermates received Rapamycin or vehicle every other day for 15 days. Rapamycin treatment reduces the expression of nuclear BMAL1 in K14cre Pten^F/F^ mice (arrowhead) (*** p<0.001) to similar levels found in control mice (ns: p>0.05). Scale bars −30 μm.

The progressive accumulation of BMAL1 and pS6 in HNSCC with compromised PTEN function suggests crosstalk between mTOR and clock signaling. We next examined the effect of mTOR on the accumulation of BMAL1. Our work and the work of others have previously shown that disruption of *PTEN* results in activation of mTOR signaling [20, 26, 29, 30]. We used Rapamycin, a well-known specific inhibitor of mTOR [31], to examine a potential role for mTOR in the accumulation of BMAL1 in K14Cre Pten^F/F^ mice. We found that vehicle-treated K14Cre Pten^F/F^ mice had an abrupt accumulation of nuclear BMAL1 compared to control littermates (Figure 4, *** p<0.001). Remarkably, administration of Rapamycin rescued BMAL1 accumulation by significantly diminishing the amount of BMAL1-positive cells in K14Cre Pten^F/F^(Figure 4) (*** p<0.001). Furthermore, rapamycin reduced BMAL1 expression in K14Cre Pten^F/F^ mice to levels comparable to control littermates (Figure 4) (ns p>0.05).

### Nuclear BMAL1 increase requires mTOR signaling *in vivo*

**Figure 5:**
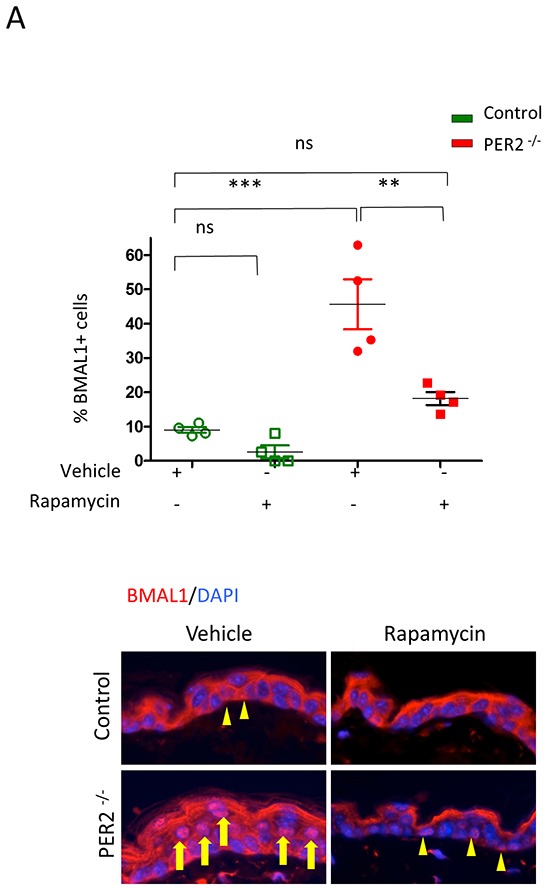
Rapamycin reduces the accumulation of BMAL1 in Per2 knockout mice **A.** As shown in the left panel, tissue samples from Per2 knockout mice (mPER^−/−^) depict robust accumulation of nuclear BMAL1 (arrow) compared to control littermates (arrowhead)(*** p<0.001). Administration of Rapamycin reduces the accumulation of BMAL1 in the epidermis of mPer^−/−^ mice (arrowhead) compared to mPer^−/−^ mice receiving vehicle alone (** p<0.01) to levels comparable to wild-type mice receiving vehicle alone (ns: p>0.05).

The circadian rhythm molecule Period2 (Per2) plays a critical role as a tumor suppressor by controlling levels of BMAL1. To confirm that BMAL1 nuclear accumulation depends on mTOR activity, we test whether mTOR inhibition interferes with PER2-driven BMAL1 accumulation. For this, we used Per2 core negative transcriptional inhibitor of the circadian clock knockout mice that harbor constitutively active BMAL1 [32–34]. Accumulation of BMAL1 in mPer2 knockout mice occurs in the epidermis (Figure 5A). BMAL1 levels were more than three-fold higher in mPer2 knockout mice than wild-type littermate controls (*** p<0.001) (Figure 5A). We showed that mPer2 knockout mice treated with Rapamycin had a significant reduction in BMAL1 expression in the epidermis compared to mPer2 mice treated with vehicle (** p<0.01), resulting in BMAL1 levels similar to WT control mice (ns p>0.05)(Figure 5A). Our data suggests that mTOR plays a critical role in the control of BMAL1.

### mTORC1 and mTORC2 are involved in regulating BMAL1 in HNSCC

**Figure 6:**
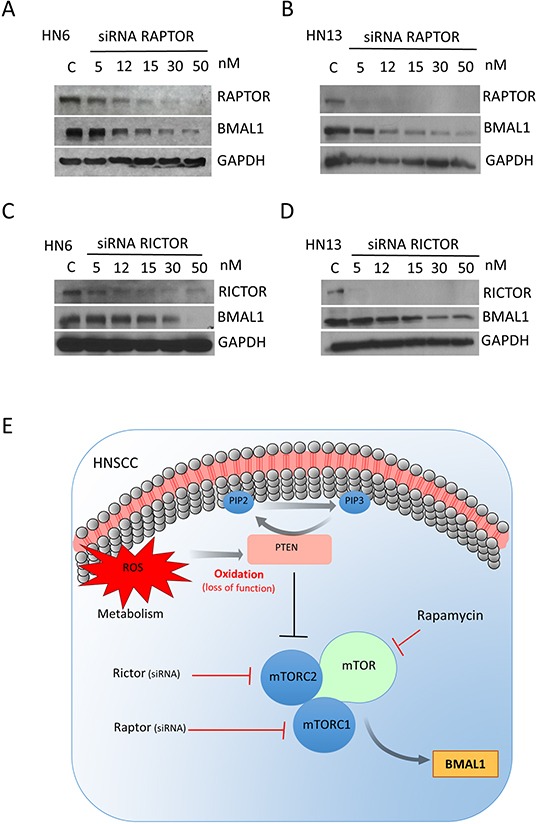
Small interference RNA targeting Raptor and Rictor disrupts BMAL1 accumulation in HNSCC Targeted disruption of Raptor **(A-B)** and Rictor **(C-D)** using siRNA results in a dose-dependent downregulation of BMAL1 in HNSCC cells. **E.** Disruption of PTEN by protein oxidation causes activation of mTOR signaling, resulting in accumulation of BMAL1. Notably, inhibition of mTOR signaling, particularly mTORC1 and mTORC2, results in restoration of normal BMAL1 levels in the epidermis of mice and head and neck cancer cells. These results demonstrate a novel role for mTOR in regulating nuclear levels of the core clock gene BMAL1.

We have shown that downregulation of the tumor suppressor gene PTEN in HNSCC results in activation of the mTOR signaling pathway [7] and accumulation of BMAL1 (Figure 3A and 3B). Using genetically defined animal models; we also showed here that inhibition of mTOR reestablished the normal expression levels of BMAL1 protein in K14Cre Pten^F/F^ and mPer2 knockout mice, suggesting a critical role for mTOR in controlling and maintaining BMAL1. We next examined whether HNSCC requires mTORC1 and mTORC2 signaling to drive BMAL1 activation. Using siRNA technology, we interfered with the mTORC1 and mTORC2 complexes by disrupting their scaffold proteins, Raptor [35, 36] and Rictor [37, 38] (Figure 6A–6D). Silencing Raptor and Rictor resulted in reduced BMAL1 expression. Overall, Raptor knockdown led to augmented downregulation of BMAL1 levels (Figure 6A and 6B) when compared to Rictor (Figure 6C and 6D). Nonetheless, small interference RNAs against Raptor and Rictor efficiently downregulated BMAL1. Collectively, our findings suggest that loss of function of PTEN in HNSCC results in constitutive accumulation of BMAL1 in an mTOR-dependent manner. Similarly, we also observed that accumulation of BMAL1 resulting from disruption of PER2 requires mTOR signaling. Our findings suggest that mTOR can act as a master regulatory mechanism of BMAL1 in normal and malignant epithelial cells (Figure 6E).

## DISCUSSION

Dysfunction in circadian rhythm is associated with an increased incidence of solid tumors. Working the night shift was linked with high prostate-specific antigen (PSA) levels and increased risk of developing prostate, as well as, breast cancers [39–43]. Indeed, increased incidence of prostate cancer was also found in individuals living in countries with high light exposure at night [44] and those who sleep less [45–47]. Breast cancer patients who experience altered sleep-wake cycles or work night shifts also have acquired resistance to Tamoxifen therapy [48]. Although the circadian rhythm clearly influences the behavior of solid tumors, a better understanding of the molecular events that control clock gene expression in cancer is needed.

The circadian rhythm is a complex molecular circuitry comprised of transcriptional activators and negative regulators with expression levels that oscillate in a day/night fashion. Positive signaling of the clock machinery is mediated largely by CLOCK and BMAL1, which induce clock-controlled genes. Other members of the clock regulatory machinery are negative feedback regulators, which are activated when CLOCK and BMAL1 are downregulated. PER2 is a member of the cryptochrome and period negative feedback regulators of the clock machinery. PER2 downregulation is present in leukemia, glioma, breast cancer [49–52]; and its deletion results in the development of lymphomas [34]. These findings suggest that *PER2* is a tumor suppressor gene, a role that has been demonstrated in mice [34]. Notably, *PER2* downregulation leads to BMAL1 activation. Indeed, we observed this activation in the epidermis of mPer2 knockout mice. Likewise, excision of PTEN, a tumor suppressor gene, unexpectedly led to the accumulation of BMAL1 in the epidermis of mice. The PTEN tumor suppressor is the major negative regulator of the PI3K/mTOR pathway, which is one of the most frequently active pathways in human cancers, including HNSCC [53–55].

We have previously demonstrated that pharmacological inhibition of the mTOR signaling using Rapamycin results in rescue of the skin phenotype observed in PTEN deficient mice [20]. Based on our previous findings, we asked whether Rapamycin would inhibit BMAL1 accumulation in PTEN conditional knockout and PER2 knockout mice. Remarkably, administration of Rapamycin blocked BMAL1 accumulation in both animal models, suggesting that mTOR serves as a key regulatory mechanism underlying epithelial circadian rhythm. Indeed, our results aligned with mTOR being directly associated with the control of organismal metabolism, similar to CLOCK and BMAL1 (reviewed in [56, 57]). Accumulation of pS6, readout of mTOR activation, in the frontal cortex, heart, and liver was also observed in *Bmal1* knockout mice [58]. BMAL1-dependent regulation of the mTOR signaling pathway also affects aging and cellular senescence [58]. We also showed that accumulation of BMAL1 requires mTOR activity *in vitro* and *in vivo*. Our findings indicate BMAL1 as a readout of compromised *PTEN* and *PER2* function and suggest that BMAL1 is involved in the progression of cancer. Likewise, if BMALl is removed, it leads to activation of senescence [58].

The correlation between the compromised function of tumor suppressors and the circadian rhythm is also poorly understood. PTEN mutations are observed in a fraction of HNSCC [3, 4], whereas the vast majority of HNSCC have functional PTEN gene but downregulated protein expression [6–8]. We found that H_2_O_2_ caused transient inactivation of PTEN protein in HNSCC. Accumulation of H_2_O_2_ is commonly observed in cancer as a by-product of deregulated metabolism, and its effects on PTEN have been shown in HeLa and NIH3T3 cells [19, 59]. Our data showed that oxidation-mediated inactivation of PTEN in HNSCC cells also leads to unexpected accumulation of the core clock protein BMAL1.

The connection between the activation of reduction/oxidation pathways and the circadian rhythm has been shown in zebrafish [60], in which the light modulates the circadian clock in Z3 light-responsive cells through the production of intracellular H_2_O_2_ that acts as an ROS signaling molecule. Notably, our data revealed that H_2_O_2_ induces activation of intracellular ROS and nuclear BMAL1 in HNSCC cells. Nevertheless, PTEN, rather than H_2_O_2_ alone, is a critical regulator of clock genes. Our results lead to meaningful findings. First, oxidation-driven and genetically defined inhibition of PTEN results in BMAL1 accumulation in normal and malignant epithelial cells. Second, PTEN-induced accumulation of BMAL1 requires a functional mTOR signaling. Third, PTEN-independent and PER2-driven accumulation of BMAL1 also requires a functional mTOR signaling. Fourth, maintenance of levels of BMAL1 in cancer cells requires functional mTORC1 and mTORC2 complexes. Although the requirements for mTOR signaling to induce BMAL1 expression in HNSCC are demonstrated in this manuscript, it's still unknown if a similar mechanism would be existent during normal epithelial homeostasis. This study is also limited in the identification of different physiological mechanisms and conditions that could lead to a transient downregulation of PTEN in HNSCC tumors. Future studies are needs to further contribute to our knowledge on how deregulated molecules contribute to HNSCC.

In summary, we showed that downregulation of PTEN leads to activation of mTOR signaling and accumulation of BMAL1. Notably, inhibition of mTOR signaling (mTORC1 and mTORC2) results in restoration of normal BMAL1 levels in the epidermis in vivo, and also in head and neck cancer cells. In addition, these results suggest that BMAL1 activation in conjunction with mTOR is required for skin phenotype found in PER2 and PTEN mice. These results demonstrated a novel role for mTOR in regulating nuclear levels of the core clock gene BMAL1.

## MATERIALS AND METHODS

### Cell lineages and reagents

HNSCC cell lines (Cal27 (tongue), WSU-HN6 (base of tongue), WSU-HN12 (lymph node/metastasis), WSU-HN13 (tongue), UMSCC17B (lymph node/metastasis), and UMSCC74A (tongue) [61–65], HeLa cervical cells [66], and NOK-SI (spontaneously immortalized normal oral keratinocytes) cells [67] were cultured in DMEM supplemented with 10% fetal bovine serum, 100 units/ml penicillin, 100 μg/ml streptomycin, and 250 ng/ml amphotericin B. Cells were maintained in a 5% CO_2_-humidified incubator at 37°C. Cells were treated with hydrogen peroxide (1.5 mM-Sigma) and Rapamycin (50 nM – LC Laboratories). Cells were synchronized with forskolin (10μM; Sigma-Aldrich, USA) before each assay. All cells were previously authenticated by PCR amplification of short tandem repeats to ensure cell identity. Cal27 and HeLa cells were acquired from the American Type Culture Collection (ATCC-Manassas).

### Immunofluorescence (IF)

Paraffin-embedded tissues were sectioned (3-5 μm), and a standard procedure was used to dewax and hydrate the tissues through graded alcohol followed by antigen retrieval and an endogenous peroxidase block [68]. IF was performed using primary antibodies BMAL1 (NB100-2288, Novus Biological, Littleton, CO), pS6 (S235/236, Cell Signaling Technology, Danvers, MA), and Ki-67 (Cell Signaling Technology, Danvers, MA) (Supplementary Table S1). After incubation overnight, slides were washed with PBS, incubated with a secondary antibody conjugated with either fluorescein (Jackson ImmunoResearch Labs 1:100) or rhodamine (Jackson Immuno Research Labs 1:100) and mounted with media containing DAPI (Vector Laboratories). Images were taken using a QImaging ExiAqua monochrome digital camera attached to a Nikon Eclipse 80i Microscope (Nikon, Melville, NY) and by a color QImaging Publisher attached to a Leica CTR5000 microscope. Images were visualized with QCapturePro software.

### ROS assay

The ROS assay was performed as previously reported [69]. Intracellular levels of ROS were analyzed using chloromethyl CM-H2DCFDA (Molecular Probes/Life Technologies, Grand Island, New York) and detection of positive cells using a Nikon fluorescent microscope. ROS was detected after intracellular esterases removed the acetate groups upon cellular oxidation.

### Experimental mice

The in vivo study was performed according to the University of Michigan Committee on Use and Care of Animals (UCUCA) approved protocol and in compliance with the Guide for the Care and Use of Laboratory Animals. Animals were housed in 12-hrs light/dark cycles, and they received standard rodent chow and water ad libitum in compliance with AAALAC guidelines. Pten^F/F^ mice (The Jackson Laboratory) were crossed with K14Cre mice to generate K14Cre Pten mutant mice [20]. Briefly, K14Cre PTEN^F/+^ mice were crossed with Pten^F/F^ mice to generate K14Cre Pten^F/F^, K14Cre Pten^F/+^ and control mice in the same litter. Genotyping was performed on tail biopsies using a PCR assay with primers previously described [20]. mPer2 knockout mice (Per2*^tm1Drw^*) were genotyped as previously described [32].

### Administration of rapamycin

Rapamycin (LC Laboratories) was reconstituted in absolute ethanol at 10 mg/mL and stored at −20°C. Rapamycin was diluted in 5.2% Tween 80 (Sigma) and 5.2% polyethylene glycol (PEG-400; Hampton Research) and injected i.p. (1 mg/kg) every other day for 15 days [20, 68]. Only vehicle solution was administered as controls.

### Western blotting

Tumor cells were lysed with cell lysis buffer containing protease inhibitors and briefly sonicated. Total protein was run in sodium dodecyl sulfate-polyacrylamide gel electrophoresis (SDS-PAGE) and transferred to an Immobilon membrane (Millipore, Billerica, MA, USA). Membranes were blocked in 5% nonfat dry milk containing 0.1 M Tris (pH 7.5), 0.9% NaCl and 0.05% Tween-20 for 1 hour at room temperature. Membranes were incubated with PTEN (Cell Signaling Technology, Danvers, MA), p-S6 (S235/236, Cell Signaling Technology, Danvers, MA), BMAL1 (NB100-2288, Novus Biological, Littleton, CO), Raptor (53A2 - Cell Signaling Technology), and Rictor (24C12 - Cell Signaling Technology) primary antibodies at 4°C overnight (Supplementary Table S1). Membranes were then incubated with appropriate secondary antibodies conjugated to horseradish peroxidase (Santa Cruz Biotechnology, Sta. Cruz, CA, USA). The signal was developed using the ECL SuperSignal West Pico Substrate (Pierce Biotechnology, Rockford, IL, USA). GAPDH served as a loading control (Calbiochem, Gibbstown, NJ, USA). Identification of reduced and oxidized forms of PTEN was performed as previously described [19]. Briefly, cells were scraped into an ice-cold solution containing 50% trichloroacetic acid and sonicated. After centrifugation, cell lysate was washed with buffer containing 0.2 ml of Tris-HCL (100 nM, PH 6.8), 2% SDS, and 40 nM of MEM followed by polyacrylamide gel electrophoresis.

### Raptor and rictor knockdown

Knockdown of Raptor and Rictor was performed as previously described [70–72]. In detail, cells were seeded in 24-well plates and transfected with HiPerFect (Qiagen) using 15 nM double-stranded RNA oligonucleotides directed against human Raptor (forward: 5′- GGA CAA CGG CCA CAA GUAdTdT-3′ and reverse: 5′- UAC UUG UGG CCG UUG UCCdTdT-3′), or 5 nM double-stranded RNA oligonucleotides against Rictor (forward: 5′- CCU AAU GAA UAU GGC UGC AUC CUU UdTdT-3′ and reverse: 5′- AAA GGA UGC AGC CAU AUU CAU UAG GdTdT-3′) (Invitrogen). Optimal concentrations and time points were determined by dilution curves of siRNA for each target and immunoblot analysis. The sequences of the control negative siRNA (Invitrogen) oligonucleotides were as follows: 5′-UUC UCC GAA CGU GUC ACG UdTdT-3′ and 5′- ACG UGA CAC GUU CGG AGA AdTdT-3′[73]. The sequences of the PTEN siRNA were 5′-CCAAUGGCUAAGUGAAGAUGACAAUdTdT-3′ and 5′-AUUGUCAUCUUCACUUAGCCAUUGGdTdT-3′.

### Statistical analysis

Statistical analyzes were performed using GraphPad Prism 5 (GraphPad Software, San Diego, CA). Statistical analyzes of positive cells for BMAL1 in K14Cre Pten and Per2 knockout mice and others were performed using one-way ANOVA followed by the Tukey's multiple comparison tests. Asterisks denote statistical significance (*p<0.05; **p<0.01; ***p<0.001; and NS p>0.05).

## SUPPLEMENTARY TABLE


