# Supplementary material for: PI3K-PTEN dysregulation leads to mTOR-driven upregulation of the core clock gene BMAL1 in normal and malignant epithelial cells
Source: Oncotarget. 2016 Jun 7;7(27):42393–407. doi: 10.18632/oncotarget.9877 (PMC5173143; doi:10.18632/oncotarget.9877)
Supplement: Supplementary file 1 [file oncotarget-07-42393-s001.pdf]

PI3K-PTEN dysregulation leads to mTOR-driven upregulation of the core clock gene BMAL1 in normal and malignant epithelial cells

SUPPLEMENTARY TABLE

Supplementary Table S1: Antibody usage

Antibody	Company	Dilution	Assay
BMAL1	Novus Biological	1:100; 1:1000	IF; WB
pS6	Cell Signaling Tech	1:100; 1:1000	IF; WB
Ki-67	Cell Signaling Tech	1:400	IF
PTEN	Cell Signaling Tech	1:1000	WB
Raptor	Cell Signaling Tech	1:1000	WB
Rictor	Cell Signaling Tech	1:1000	WB

IF- immunofluorescence; WB- western blot
